# Association of decreased CYP2C9 function, HLA-B*40:06, and hemoglobin levels with phenytoin-induced cutaneous adverse reactions in a Southern Thai population

**DOI:** 10.3389/fphar.2026.1827880

**Published:** 2026-07-13

**Authors:** Chutima Seree-aphinan, Antida Sangiemchoey, Suwanna Setthawatcharawanich, Sutthiporn Pattharachayakul, Areerat Hnoonual, Warit Ruanglertboon, Porntip Intapiboon, Anukoon Kaewborisutsakul, Titaporn Thamcharoenvipas, Pornruedee Rachatawiriyakul, Tabtim Chongsuvivatwong, Sirianong Sittipong, Chonlaphat Sukasem, Kanoot Jaruthamsophon

**Affiliations:** 1 Department of Internal Medicine, Faculty of Medicine, Prince of Songkla University, Hat Yai/Songkhla, Thailand; 2 Division of Pharmacy, Songklangarind Hospital, Faculty of Medicine, Prince of Songkla University, Hat Yai/Songkhla, Thailand; 3 Department of Clinical Pharmacy, Faculty of Pharmaceutical Sciences, Prince of Songkla University, Hat Yai/Songkhla, Thailand; 4 Department of Pathology, Faculty of Medicine, Prince of Songkla University, Hat Yai/Songkhla, Thailand; 5 Department of Pharmacology, Faculty of Science, Prince of Songkla University, Hat Yai/Songkhla, Thailand; 6 Neurological Surgery Unit, Department of Surgery, Faculty of Medicine, Prince of Songkla University, Hat Yai, Songkhla, Thailand; 7 Department of Pediatrics, Faculty of Medicine, Prince of Songkla University, Songkhla, Thailand; 8 Thonburi Rajyindee Hospital, Hat Yai/Songkhla, Thailand; 9 Division of Neurology, Department of Medicine, Hat Yai Medical Education Center, Hat Yai, Songkhla, Thailand; 10 Songkhla Hospital, Songkhla, Thailand; 11 Department of Pathology, Faculty of Medicine Ramathibodi Hospital, Mahidol University, Bangkok, Thailand; 12 Faculty of Pharmaceutical Sciences, Burapha University, Mueang, Chonburi, Thailand

**Keywords:** drug allergy, drug eruptions, pharmacogenetics, pharmacological biomarkers, precision medicine

## Abstract

**Background:**

Phenytoin is an anticonvulsant used to treat epilepsy and prevent perioperative seizures. However, genetic prediction of phenytoin-induced cutaneous adverse reactions (PHT-cADRs) and the co-contributing role of non-genetic factors remain unclear. We aimed to evaluate genetic and non-genetic factors associated with PHT-cADRs in a Southern Thai population.

**Methods:**

A case–control study was conducted involving 104 patients (26 with PHT-cADRs confirmed by a dermatologist or allergist and 78 phenytoin-tolerant controls). Patient history (demographics, comorbidities, and co-medications) and laboratory parameters (complete blood count and clinical chemistry indicators) during phenytoin initiation were reviewed. Genetic markers including *CYP2C9*, *HLA-A*, *HLA-B*, and *HLA-DRB1* were studied.

**Results:**

A significant difference in sex was observed among the patients (73.1% female patients in the PHT-cADR group vs. 44.9% in the control group; P = 0.014). Baseline clinical indications differed marginally between the groups; the case group had more neurosurgical patients and fewer patients with epilepsy than the control group. Other non-genetic factors were not significantly associated with PHT-cADRs. For laboratory data, the case group showed significantly lower mean hemoglobin (11.73 g/dL vs. 13.48 g/dL, P = 0.0005) and hematocrit levels (35.94% vs. 40.29%, P = 0.0019) and a higher mean white blood cell count (10.19 × 10^3^/μL vs. 7.75 × 10^3^/μL, P = 0.0083) than the control group. For genetic factors, decreased CYP2C9 function [odds ratio (OR) = 7.89; 95% confidence interval (CI) = 1.81–34.49; P = 0.006] and HLA-B*40:06 (OR = 6.26; 95% CI = 1.59–24.69; P = 0.009) were associated with an increased risk of PHT-cADRs. No HLA marker reached a significant level after Bonferroni correction. Multivariate analysis was not performed owing to a high proportion of missing laboratory data.

**Conclusion:**

*HLA-B* alleles and CYP2C9 function were associated with PHT-cADRs. As an exploratory observation, our findings suggest that lower hemoglobin levels and higher white blood cell counts are associated with PHT-cADRs. However, the limited sample size, particularly in the case group, may have led to false-positive and -negative results. Therefore, these findings should be interpreted with caution, and a larger study is required to confirm the contribution of these hematological factors.

## 1 Introduction

Phenytoin is an anticonvulsant commonly used to treat epilepsy and to prevent perioperative and post-traumatic seizures in patients with traumatic brain injury (Huo et al., 2022). Despite its utility, phenytoin-induced cutaneous adverse reactions (PHT-cADRs) remain a major challenge in clinical settings. The spectrum of PHT-cADRs ranges from relatively mild maculopapular exanthema to potentially fatal conditions, such as drug reactions with eosinophilia and systemic symptoms (DRESS) and Stevens–Johnson syndrome (SJS).

To mitigate these risks, the Clinical Pharmacogenetics Implementation Consortium recommends testing for HLA-B*15:02 and *CYP2C9* genotypes before prescribing phenytoin (Karnes et al., 2021). However, the pharmacogenetics of phenytoin remain unclear. Several studies have reported a negative association between HLA-B*15:02 and PHT-cADRs, along with positive associations with alternative HLA markers, including HLA-A*24:02, HLA-B*40:01, HLA-B*51:01, and HLA-B*56:02 (Su et al., 2019; Fohner et al., 2020; Manuyakorn et al., 2020; Sukasem et al., 2020; John et al., 2021)*.* Among drug-metabolizing enzymes, decreased CYP2C9 function is associated with an increased risk of phenytoin toxicity and PHT-cADRs (Chung et al., 2014; Suvichapanich et al., 2015; Tassaneeyakul et al., 2016; Yampayon et al., 2017; Fohner et al., 2019; Su et al., 2019; Hikino et al., 2020; Sukasem et al., 2020; John et al., 2021). However, reports on the successful implementation of phenytoin pharmacogenetic markers are limited (Chang et al., 2020). Additionally, a systematic review in 2024 reported higher phenytoin concentrations among those with *CYP2C19* intermediate and poor metabolizer phenotypes than among those with the extensive metabolizer phenotype (Milosavljević et al., 2024). This highlights the complexity of the pharmacogenetic mechanisms underlying the pharmacological effects of phenytoin.

In addition to genetic predisposition, the influence of non-genetic factors on PHT-cADRs is complex. Because phenytoin forms haptens with human serum albumin, it is required to monitor unbound phenytoin levels in patients with hypoalbuminemia and hepatic and renal diseases (Food and Drug Administration, 2021). Furthermore, owing to the non-linear pharmacokinetics of phenytoin, other undiscovered variables have been hypothesized to affect free drug levels, making plasma concentration difficult to predict (Montgomery et al., 2019; Ter Heine et al., 2019). Clinical factors, such as omeprazole co-administration, have been reported as independent risk factors for severe PHT-cADRs (Yampayon et al., 2017). These diverse effects make it difficult to comprehensively understand phenytoin hypersensitivity.

In this study, we aimed to conduct a case–control study in a Southern Thai population to simultaneously evaluate the roles of genetic and non-genetic factors in PHT-cADRs. Clinical information for each patient was retrieved from the Hospital Information System. Genotyping of *HLA-A*, *HLA-B*, and *HLA-DRB1* was performed using polymerase chain reaction–sequence-specific oligonucleotide probing. Genotyping of *CYP2C9* and *CYP2C19* was performed using matrix-assisted laser desorption/ionization time-of-flight (MALDI-TOF) mass spectrometry. Clinical and laboratory data were analyzed, along with genetic studies, to evaluate the interplay between these factors.

## 2 Materials and methods

### 2.1 Participants

This retrospective case–control study included 104 participants (26 patients with PHT-cADRs and 78 phenytoin-tolerant controls) from the Songklanagarind Hospital. Eligible participants were aged between 18 and 70 years and self-reported to originate from Southern Thailand. Participants were enrolled between October 2024 and May 2025. The clinical diagnoses in the PHT-cADR group included maculopapular rash (n = 21), DRESS (n = 3), and SJS (n = 2).

Patients with PHT-cADRs diagnosed or confirmed by a dermatologist or allergist were included in this study. Cases without previous specialist confirmation were retrospectively reassessed by a dermatologist before enrollment. Causality was assessed using the Naranjo probability scale, and only those with probable or certain scores were included. Hypersensitivity cases in which the culprit drug was unclear (Naranjo probability scale of possible or doubtful) were excluded. The tolerant control group included patients who received phenytoin continuously for at least 90 days without any adverse skin reactions. The age was matched between the groups. Patients diagnosed with human immunodeficiency virus infection at the time of phenytoin prescription and those who were pregnant at the time of recruitment were excluded.

### 2.2 Data retrieval

Clinical and laboratory data on phenytoin initiation were retrieved and reviewed using the Hospital Information System. The collected clinical data included demographics, first date of phenytoin prescription, last date of phenytoin prescription, starting dose of phenytoin, comorbidities, and co-medication(s). Ethnicity was determined based on self-reported data recorded in the Hospital Information System. The collected baseline laboratory data (before starting phenytoin) included complete blood counts (white blood cell [WBC] count, red blood cell [RBC] count, hemoglobin level, hematocrit level, and platelet count) and clinical chemistry parameters (albumin, total protein, aspartate aminotransferase [AST], alanine aminotransferase [ALT], alkaline phosphatase, blood urea nitrogen [BUN], and creatinine levels). Anemia status was interpreted based on the sex-specific cutoff of the hemoglobin level (12.5 g/dL for female patients and 13.5 g/dL for male patients). The study protocol was approved by the local Ethics Committee of the Faculty of Medicine, Prince of Songkla University (REC 67-328-5-1), and the Central Research Ethics Committee of Thailand (CREC011/67BR-BIO2).

### 2.3 Genetic testing

Genotyping of *CYP2C9* and *CYP2C19* was performed at the Molecular Diagnostics Laboratory of Songklanagarind Hospital using MALDI-TOF mass spectrometry (MassARRAY; Agena Bioscience, CA, United States). This assay targeted common variants in the Thai population, including CYP2C9*2 (rs1799853), CYP2C9*3 (rs1057910), CYP2C19*2 (rs4244285), CYP2C19*3 (rs4986893), and CYP2C19*17 (rs 12248560). Genotyping of *HLA-A*, *HLA-B*, and *HLA-DRB1* was performed at the Pharmacogenetic Laboratory, Ramathibodi Hospital, using Lifecodes HLA SSO typing kits and analyzed using the Luminex® IS100 system (Austin, TX, United States).

### 2.4 Statistical analysis

Statistical associations between clinical factors, laboratory parameters, genetic markers, and PHT-cADRs were evaluated using the chi-square test, Mann–Whitney U test, or Fisher’s exact test, as appropriate. Allele and genotype frequencies of the case and control groups were compared to determine odds ratios (ORs), 95% confidence intervals (CIs), and P-values. To account for multiple comparisons in the HLA marker analysis, corrected P-values (Pc) were calculated using the Bonferroni correction. Statistical significance was set at P < 0.05.

## 3 Results

### 3.1 Baseline characteristics

The demographics and characteristics of the participants are presented in Table 1. The case group had a significantly higher proportion of female participants than the control group (P = 0.014). The mean ages of the case and control groups were 48.5 and 48.7 years, respectively. No significant differences were observed between the groups in terms of body weight, body height, body mass index, ethnicity, phenytoin indication, comorbidity, or co-medication (Table 1). For laboratory parameters, missing data were less than 50% in both groups for all hematological parameters (hemoglobin, hematocrit, WBC count, platelet count, and RBC count), BUN, and creatinine, but not for the liver function profile (alkaline phosphatase, AST, ALT, total protein, albumin, total bilirubin, and direct bilirubin) (Table 1). A comparison of the baseline laboratory profiles showed significantly lower hemoglobin and hematocrit levels and a higher WBC count in the case group than in the control group (Table 1). No significant difference was observed in the RBC counts between the groups. The WBC count was significantly higher in the case group than in the control group. Among the clinical chemistry parameters, only alkaline phosphatase levels were significantly different between the groups, being higher in the tolerant group than in the case group.

**TABLE 1:** Characteristics of participants.

Characteristics	Case group (n = 26)	Tolerant group (n = 78)	P-value
Age (mean, SD)	48.5 (16.5)	48.7 (18.7)	0.968^a^
Body weight (mean, SD)	57.5 (11.8)	61.5 (15.4)	0.258^a^
Body height (mean, SD)	158.5 (6.8)	162.1 (8.2)	0.051^a^
Body mass index (mean, SD)	22.9 (4.9)	23.2 (4.5)	0.807^a^
Sex (n, %)	​	​	0.014^b^
Female	19 (73.1%)	35 (44.9%)	
Male	7 (26.9%)	43 (55.1%)	
Ethnicity (n, %)	​	​	0.463^c^
Thai Buddhist	23 (88.5%)	63 (80.8%)	
Thai Muslim	2 (7.7%)	12 (15.4%)	
Thai Chinese	1 (3.8%)	3 (3.8%)	
Phenytoin indication (n, %)	​	​	​
Epilepsy	12 (46.2%)	57 (73.1%)	0.054^c^
Neurosurgery	12 (46.2%)	16 (20.5%)	
Traumatic brain injury	1 (3.8%)	4 (5.1%)	
Trigeminal neuralgia	1 (3.8%)	1 (1.3%)	
Latency period (mean, SD)	-	13.4 (8.3)	-
Smoking (n, %)	2 (7.69%)	8 (10.26%)	1.000^b^
Alcohol consumption (n, %)	3 (11.54%)	5 (6.41%)	0.409^b^
Comorbidity (n,%)	​	​	​
Obesity	7 (28.0%)	23 (29.5%)	1.000^b^
Cancer	3 (11.5%)	16 (20.5%)	0.390^b^
Hypertension	5 (19.23%)	12 (15.38%)	0.540^b^
Diabetes mellitus type II	5 (19.23%)	6 (7.69%)	0.124^b^
Stroke	3 (11.54%)	13 (16.67%)	0.652^b^
Dyslipidemia	4 (15.38%)	17 (21.79%)	0.582^b^
Systemic lupus erythematosus	1 (3.9%)	2 (2.6%)	1.000^b^
Comedication (n, %)	​	​	​
Omeprazole (n, %)	5 (19.2%)	9 (11.5%)	0.332^b^
Antibiotic (n, %)	3 (11.5%)	6 (7.7%)	0.687^b^
Other anticonvulsant (n, %)	13 (50.0%)	47 (60.3%)	0.371^b^
NSAIDs (n, %)	1 (3.9%)	4 (5.1%)	1.000^b^
Simvastatin (n, %)	3 (11.5%)	9 (11.5%)	1.000^b^
Metformin (n, %)	2 (7.7%)	3 (3.9%)	0.597^b^
Losartan (n, %)	1 (3.9%)	4 (5.1%)	1.000^b^
Amlodipine (n, %)	1 (3.9%)	2 (2.6%)	1.000^b^
Atorvastatin (n, %)	0 (0.0%)	9 (11.5%)	0.108^b^
Enalapril (n, %)	0 (0.0%)	3 (3.9%)	0.571^b^
Clopidogrel (n, %)	0 (0.0%)	3 (3.9%)	0.571^b^
Hematologic parameters	​	​	​
Hemoglobin (g/dL)	​	​	​
Tested (%)	22 (84.6%)	50 (64.1%)	0.0542^b^
Mean (SD)	11.73 (1.76)	13.40 (2.07)	0.0005^a^
Hematocrit (%)	​	​	​
Tested (%)	22 (84.6%)	50 (64.1%)	0.0542^b^
Mean (SD)	35.94 (5.20)	40.29 (5.72)	0.0019^a^
White blood cell (WBC) count (×10^3^/μL)	​	​	​
Tested (%)	22 (84.6%)	50 (64.1%)	0.0542^b^
Mean (SD)	10.19 (3.92)	7.75 (2.21)	0.0083^a^
Platelet count (×10^3^/μL)	​	​	​
Tested	22 (84.6%)	48 (61.5%)	0.0322^b^
Mean (SD)	257.4 (102.0)	260.5 (84.9)	0.6172^a^
Red blood cell (RBC) count (×10^6^/μL)			
Tested	22 (84.6%)	47 (60.3%)	0.0303^b^
Mean (SD)	4.56 (0.93)	4.56 (0.76)	0.6155^a^
Anemic*			
Yes	15 (68.2%)	18 (36.0%)	0.0199^b^
No	7 (31.8%)	32 (64.0%)	
Clinical Chemistry	​	​	​
Creatinine (mg/dL)	​	​	​
Tested (%)	21 (80.8%)	46 (59.0%)	
Mean (SD)	1.07 (1.19)	1.38 (2.34)	0.2315^a^
Blood urea nitrogen (BUN) (mg/dL)			
Tested (%)	19 (73.1%)	40 (51.3%)	
Mean (SD)	14.39 (8.49)	15.44 (14.67)	0.7827^a^
Liver function test	​	​	​
Alkaline phosphatase (U/L)	​	​	​
Tested (%)	14 (53.8%)	33 (42.3%)	​
Mean (SD)	77.21 (50.83)	98.64 (58.40)	0.0384^a^
Aspartate aminotransferase (AST) (U/L)	​	​	​
Tested (%)	14 (53.8%)	36 (46.2%)	
Mean (SD)	307.07 (1018.5) **	24.72 (10.41)	0.7049^a^
Alanine aminotransferase (ALT) (U/L)	​	​	
Tested (%)	14 (53.8%)	36 (46.2%)	
Mean (SD)	84.36 (155.09)**	27.75 (14.31)	0.3811^a^
Total protein (g/L)	​	​	
Tested (%)	14 (53.8%)	30 (38.5%)	
Mean (SD)	7.06 (0.81)	7.52 (0.73)	0.0793^a^
Albumin (g/L)	​	​	
Tested (%)	16 (61.5%)	37 (47.4%)	
Mean (SD)	4.1 (0.55)	4.3 (0.53)	0.1926^a^
Total bilirubin (mg/dL)			
Tested (%)	14 (53.8%)	30 (38.5%)	
Mean (SD)	0.80 (1.70)	0.40 (0.26)	0.8997^a^
Direct bilirubin (mg/dL)			
Tested (%)	14 (53.8%)	29 (37.2%)	
Mean (SD)	0.50 (1.29)	0.16 (0.08)	0.9482^a^

NSAIDs, non-steroidal anti-inflammatory drugs; SD, standard deviation; U/L, units per liter.

*Hb lower than sex-specific cut-off (female: 12.5 g/dL, male: 13.5 g/dL), ** data containing an outlier.

^a^ Mann–Whitney U test,

^b^ Fisher’s exact test.

^c^ Chi-square test.

### 3.2 Genetic association

Genetic analyses of *CYP2C9* and *CYP2C19* using MassARRAY genotyping were performed on 103 samples (25 cases and 78 controls) (Table 2). Decreased CYP2C9 function and the CYP2C9*1/*3 genotype were associated with an increased risk of PHT-cADRs (P = 0.006 and P = 0.015, respectively). The proportions of patients with decreased CYP2C9 function were 24.0% (6/25) and 3.8% (3/78) in the case and control groups, respectively. In addition, the CYP2C19*1/*3 genotype was more frequent in the case group than in the tolerant group (3/25 vs. 1/78; P = 0.018). However, decreased CYP2C19 function was not associated with an increased risk of developing PHT-cADRs (Table 2).

**TABLE 2:** Comparison of *CYP2C9* and *CYP2C19* between the groups.

Characteristics	Case group (n = 25)	Tolerant group (n = 78)	Odds ratio (95% CI)	P-value
*CYP2C9* function	​	​	​	​
Decreased function	6 (24.0%)	3 (3.8%)	7.89 (1.81–34.49)	0.006
Normal function	19 (76.0%)	75 (96.2%)		
*CYP2C19* function	​	​	​	​
Decreased function	16 (64.0%)	37 (47.4%)	1.97 (0.78–4.99)	0.153
Normal or increased function	9 (36.0%)	41 (52.6%)		
CYP2C9 genotype	​	​	​	​
*1/*1 (NM)	19 (76.0%)	75 (96.2%)	-	​
*1/*2 (IM, AS = 1.5)	1 (4.0%)	0 (0.0%)	11.62 (0.46–296.33)	0.138
*1/*3 (IM, AS = 1.0)	5 (20.0%)	3 (3.8%)	6.58 (1.44–30.00)	0.015
CYP2C19 genotype	​	​	​	​
*1/*1 (NM)	6 (24.0%)	37 (47.4%)	-	​
*1/*2 (IM)	11 (44.0%)	27 (34.6%)	2.51 (0.83–7.64)	0.104
*1/*3 (IM)	3 (12.0%)	1 (1.3%)	18.50 (1.64–208.47)	0.018
*2/*2 (PM)	1 (4.0%)	6 (7.7%)	1.03 (0.10–10.11)	0.981
*2/*3 (PM)	0 (0.0%)	2 (2.6%)	1.15 (0.05–26.89)	0.929
*2/*17 (IM)	1 (4.0%)	1 (1.3%)	6.17 (0.34–112.41)	0.219
*1/*17 (RM)	3 (12.0%)	4 (5.1%)	4.63 (0.82–26.03)	0.082
*CYP2C19 allele*	​	​	​	​
*2 carrier	13 (52.0%)	36 (46.2%)	1.26 (0.51–3.12)	0.611
*3 carrier	3 (12.0%)	3 (3.8%)	6.17 (1.00–37.98)	0.049
*17 carrier	4 (16.0%)	5 (6.4%)	2.78 (0.68–11.29)	0.153

AS, activity score; NM, normal metabolizer; IM, intermediate metabolizer; PM, poor metabolizer; RM, rapid metabolizer

Genetic analyses of *HLA-A*, *HLA-B*, and *HLA-DRB1* were performed via Luminex genotyping of 98 samples of adequate quality (including 23 cases and 75 controls). The genotyping results are summarized in Table 3. A total of 85 different alleles were identified (20 *HLA-A*, 41 *HLA-B*, and 24 *HLA-DRB1*). Only HLA-B*40:06 was significantly associated with an increased risk of PHT-cADRs (P = 0.009, OR = 6.26, 95% CI = 1.59–24.69). HLA-DRB1*12:02 was associated with a decreased risk of PHT-cADRs (P = 0.049, OR = 0.22, 95% CI = 0.05–1.00). No Pc value reached a significance of 0.05 after Bonferroni correction for multiple comparisons (number of tested hypotheses = 85). In addition, due to the high proportion of missing data on laboratory parameters, a multivariate analysis, including genetic and non-genetic factors, was not performed.

**TABLE 3:** *HLA-A*, *HLA-B*, and *HLA-DRB1* carrier frequency of cases and tolerant controls.

HLA status	Carrier frequency		P-value	Odds ratio (95% CI)
	Case group (n = 23)	Tolerant group (n = 75)		
HLA-A*01:01	2 (8.7%)	5 (6.7%)	0.742	1.33 (0.24–7.38)
HLA-A*02:01	5 (21.7%)	12 (16.0%)	0.526	1.46 (0.45–4.69)
HLA-A*02:03	4 (17.4%)	11 (14.7%)	0.751	1.22 (0.3496–4.2912
HLA-A*02:06	3 (13.0%)	2 (2.7%)	0.073	5.48 (0.86–35.04)
HLA-A*02:11	0 (0.0%)	4 (5.3%)	0.473	0.34 (0.02–6.52)
HLA-A*03:01	0 (0.0%)	1 (1.3%)	0.973	1.06 (0.04–26.82)
HLA-A*03:02	2 (8.7%)	0 (0.0%)	0.068	17.56 (0.81–379.75)
HLA-A*11:01	9 (39.1%)	35 (46.7%)	0.526	0.73 (0.28–1.90)
HLA-A*24:02	2 (8.7%)	14 (18.7%)	0.270	0.42 (0.09–1.98)
HLA-A*24:03	1 (4.3%)	1 (1.3%)	0.398	3.36 (0.20–56.01)
HLA-A*24:07	3 (13.0%)	11 (14.7%)	0.846	0.87 (0.22–3.44)
HLA-A*24:10	1 (4.3%)	5 (6.7%)	0.687	0.64 (0.07–5.74)
HLA-A*26:01	1 (4.3%)	3 (4.0%)	0.941	1.09 (0.11–11.02)
HLA-A*29:01/02	0 (0.0%)	3 (4.0%)	0.592	0.44 (0.02–8.85)
HLA-A*30:01	2 (8.7%)	1 (1.3%)	0.118	7.05 (0.61–81.58)
HLA-A*31:01	0 (0.0%)	1 (1.3%)	0.973	1.06 (0.04–26.82)
HLA-A*32:01	0 (0.0%)	3 (4.0%)	0.592	0.44 (0.02–8.85)
HLA-A*33:01/03	6 (26.1%)	25 (33.3%)	0.515	0.71 (0.25–2.01)
HLA-A*34:01/05	0 (0.0%)	2 (2.7%)	0.765	0.63 (0.03–13.50)
HLA-A*68:01	0 (0.0%)	2 (2.7%)	0.765	0.63 (0.03–13.50)
HLA-B*07:02/10	0 (0.0%)	1 (1.3%)	0.973	1.06 (0.04–26.82)
HLA-B*07:05/06	0 (0.0%)	6 (8.0%)	0.319	0.23 (0.01–4.19)
HLA-B*13:01	3 (13.0%)	12 (16.0%)	0.731	0.79 (0.20–3.07)
HLA-B*13:02	2 (8.7%)	1 (1.3%)	0.118	7.05 (0.61–81.58)
HLA-B*15:02	1 (4.3%)	14 (18.7%)	0.128	0.20 (0.03–1.60)
HLA-B*15:07	1 (4.3%)	0 (0.0%)	0.162	10.07 (0.40–255.8)
HLA-B*15:11	0 (0.0%)	2 (2.7%)	0.765	0.63 (0.03–13.50)
HLA-B*15:13	1 (4.3%)	4 (5.3%)	0.851	0.81 (0.09–7.60)
HLA-B*15:17	0 (0.0%)	2 (2.7%)	0.765	0.63 (0.03–13.50)
HLA-B*15:18	0 (0.0%)	2 (2.7%)	0.765	0.63 (0.03–13.50)
HLA-B*15:21	0 (0.0%)	3 (4.0%)	0.592	0.44 (0.02–8.85)
HLA-B*15:25	0 (0.0%)	4 (5.3%)	0.473	0.34 (0.02–6.52)
HLA-B*15:27	0 (0.0%)	1 (1.3%)	0.973	1.06 (0.04–26.82)
HLA-B*18:01	2 (8.7%)	5 (6.7%)	0.742	1.33 (0.24–7.38)
HLA-B*18:02	1 (4.3%)	4 (5.3%)	0.851	0.81 (0.09–7.60)
HLA-B*27:03/05	0 (0.0%)	1 (1.3%)	0.973	1.06 (0.04–26.82)
HLA-B*27:04	0 (0.0%)	1 (1.3%)	0.973	1.06 (0.04–26.82)
HLA-B*27:04/06	0 (0.0%)	1 (1.3%)	0.973	1.06 (0.04–26.82)
HLA-B*27:06	0 (0.0%)	2 (2.7%)	0.765	0.63 (0.03–13.50)
HLA-B*35:01	1 (4.3%)	4 (5.3%)	0.851	0.81 (0.09–7.60)
HLA-B*35:03	1 (4.3%)	3 (4.0%)	0.941	1.09 (0.11–11.02)
HLA-B*35:05	3 (13.0%)	4 (5.3%)	0.224	2.66 (0.55–12.89)
HLA-B*35:08	0 (0.0%)	1 (1.3%)	0.973	1.06 (0.04–26.82)
HLA-B*37:01	2 (8.7%)	2 (2.7%)	0.227	3.48 (0.45–26.18)
HLA-B*38:02	1 (4.3%)	2 (2.7%)	0.681	1.66 (0.14–19.2)
HLA-B*40:01	4 (17.4%)	12 (16.0%)	0.875	1.11 (0.32–3.83)
HLA-B*40:02	0 (0.0%)	1 (1.3%)	0.973	1.06 (0.04–26.82)
HLA-B*40:06	6 (26.1%)	4 (5.3%)	0.009	6.26 (1.59–24.69)
HLA-B*44:02	0 (0.0%)	1 (1.3%)	0.973	1.06 (0.04–26.82)
HLA-B*44:03	2 (8.7%)	15 (20.0%)	0.224	0.38 (0.08–1.81)
HLA-B*46:01	5 (21.7%)	6 (8.0%)	0.079	3.19 (0.87–11.67)
HLA-B*48:01	0 (0.0%)	1 (1.3%)	0.973	1.06 (0.04–26.82)
HLA-B*49:01	0 (0.0%)	1 (1.3%)	0.973	1.06 (0.04–26.82)
HLA-B*51:01	1 (4.3%)	4 (5.3%)	0.851	0.81 (0.09–7.60)
HLA-B*51:06	0 (0.0%)	1 (1.3%)	0.973	1.06 (0.04–26.82)
HLA-B*52:01	2 (8.7%)	4 (5.3%)	0.560	1.69 (0.29–9.88)
HLA-B*54:01	1 (4.3%)	0 (0.0%)	0.162	10.07 (0.40–255.8)
HLA-B*55:01	0 (0.0%)	0 (0.0%)	-	-
HLA-B*55:02	1 (4.3%)	1 (1.3%)	0.398	3.36 (0.20–56.01)
HLA-B*56:01	0 (0.0%)	1 (1.3%)	0.973	1.06 (0.04–26.82)
HLA-B*56:02	0 (0.0%)	0 (0.0%)	-	-
HLA-B*57:01	1 (4.3%)	2 (2.7%)	0.685	1.66 (0.14–19.18)
HLA-B*58:01	3 (13.0%)	11 (14.7%)	0.846	0.87 (0.22–3.44)
HLA-DRB1*01:01	0 (0.0%)	2 (2.7%)	0.765	0.63 (0.03–13.50)
HLA-DRB1*03:01	2 (8.7%)	8 (10.7%)	0.785	0.80 (0.16–4.05)
HLA-DRB1*04:01	1 (4.3%)	0 (0.0%)	0.162	10.07 (0.40–255.8)
HLA-DRB1*04:03	1 (4.3%)	2 (2.7%)	0.685	1.66 (0.14–19.18)
HLA-DRB1*04:04	0 (0.0%)	1 (1.3%)	0.973	1.06 (0.04–26.82)
HLA-DRB1*04:05	1 (4.3%)	2 (2.7%)	0.685	1.66 (0.14–19.18)
HLA-DRB1*04:06	0 (0.0%)	3 (4.0%)	0.592	0.44 (0.02–8.85)
HLA-DRB1*07:01	5 (21.7%)	20 (26.7%)	0.636	0.76 (0.25–2.33)
HLA-DRB1*08:03	3 (13.0%)	4 (5.3%)	0.224	2.66 (0.55–12.89)
HLA-DRB1*09:01	4 (17.4%)	10 (13.3%)	0.628	1.37 (0.39–4.86)
HLA-DRB1*10:01	3 (13.0%)	4 (5.3%)	0.224	2.66 (0.55–12.89)
HLA-DRB1*11:01	1 (4.3%)	3 (4.0%)	0.941	1.09 (0.11–11.02)
HLA-DRB1*11:06	1 (4.3%)	1 (1.3%)	0.398	3.36 (0.20–56.01)
HLA-DRB1*12:01	0 (0.0%)	1 (1.3%)	0.973	1.06 (0.04–26.82)
HLA-DRB1*12:02	2 (8.7%)	23 (30.7%)	0.049	0.22 (0.05–1.00)
HLA-DRB1*13:01	0 (0.0%)	4 (5.3%)	0.473	0.34 (0.02–6.52)
HLA-DRB1*13:02	2 (8.7%)	6 (30.7%)	0.915	1.10 (0.21–5.84)
HLA-DRB1*13:12	2 (8.7%)	1 (1.3%)	0.118	7.05 (0.61–81.58)
HLA-DRB1*14:01	3 (13.0%)	5 (6.7%)	0.337	2.10 (0.46–9.56)
HLA-DRB1*14:04	2 (8.7%)	6 (8.0%)	0.915	1.10 (0.21–5.84)
HLA-DRB1*14:10	0 (0.0%)	1 (1.3%)	0.973	1.06 (0.04–26.82)
HLA-DRB1*15:01	5 (21.7%)	19 (25.3%)	0.726	0.82 (0.27–2.51)
HLA-DRB1*15:02	4 (17.4%)	11 (14.7%)	0.751	1.22 (0.35–4.29)
HLA-DRB1*16:02	2 (8.7%)	12 (16.0%)	0.389	0.50 (0.10–2.42)

## 4 Discussion

Our study provides an exploratory analysis of the complex relationship between genetic and non-genetic factors that contribute to the development of PHT-cADRs. We identified a significant association between the *HLA-B* marker, decreased CYP2C9 function, and the risk of developing PHT-cADRs, which is consistent with the results of several previous studies (Chung et al., 2014; Suvichapanich et al., 2015; Tassaneeyakul et al., 2016; Yampayon et al., 2017; Su et al., 2019; Fohner et al., 2020; Hikino et al., 2020; Sukasem et al., 2020; John et al., 2021). We identified a patient with the CYP2C9*1/*2 genotype, which has not been previously documented in a PHT-cADR cohort from the Thai population.

HLA-B*40:06 increased the risk of PHT-cADRs, similar to the observation in a previous study in which the closely related marker, HLA-B*40:01, was positively associated with PHT-cADRs (Sukasem et al., 2020). However, despite sharing the field-1 nomenclature, these alleles belong to different serotype subfamilies. HLA-B*40:01 is the split antigen serotype B60, whereas HLA-B*40:06 is the split antigen serotype B61. HLA associations are often observed at a relatively higher frequency in populations. The frequency of HLA-B60 (HLA-B*40:01) in the Central Thai population is significantly higher than that in the Southern Thai population; in contrast, the frequency of HLA-B*40:06 is reportedly higher in the Southern than in the Central Thai population (Satapornpong et al., 2020; Kusolthammarat et al., 2025). As this study exclusively included participants from Southern Thailand, which has a unique genetic admixture of HLA genotypes and haplotypes, this regional divergence could significantly interfere with the importance of pharmacogenetic markers in these populations (Kusolthammarat et al., 2025).

We observed no significant association with other previously reported markers that are relatively common, namely, HLA-A*24:02, HLA-B*15:02, HLA-B*51:01, HLA-B*55:01, and HLA-B*56:02 (Hikino et al., 2020; Sukasem et al., 2020; Sukasem et al., 2021; Rashid et al., 2022). Because the frequencies of these markers were minimal, their associations may have been undetectable owing to the limited statistical power of the study. From a biological perspective, this suggests that conventional allele-level association studies may not fully capture the complex mechanisms underlying cADRs. Alternative approaches to allele definition may be required to gain better insights. For example, the concept of shared peptide-binding specificity among HLA molecules sheds light on the mechanisms underlying hypersensitivity reactions induced by cotrimoxazole and nevirapine (Pavlos et al., 2017; Li et al., 2025). Furthermore, the association with the HLA-B75 serotype expands our understanding of HLA-B*15:02-associated carbamazepine-induced cADR in Asian populations (Jaruthamsophon et al., 2017; Yuliwulandari et al., 2017; Capule et al., 2020; Capule et al., 2021; Nakkam et al., 2022).

As a major and novel finding of this study, positive associations were identified between PHT-cADRs and lower hemoglobin levels and elevated WBC counts. Patients in the case group had significantly lower hemoglobin and hematocrit levels than those in the control group. As the mechanism behind this novel finding is crucial, we hypothesized that this may indicate an unknown role of hemoglobin adducts in the non-linear pharmacokinetics of phenytoin, in addition to the traditional understanding of drug–albumin binding (Richens, 1979). Phenytoin is well known for its high binding affinity to serum albumin and its ability to be distributed into RBCs (Gibberd and Webley, 1975; Richens, 1979). Although hypoalbuminemia is recognized to increase the free fraction of phenytoin and the risk of toxicity (Food and Drug Administration, 2021), little is known about the ability of the drug to form adducts with hemoglobin. As a proposed explanation for the association with lower hemoglobin levels, it is possible that phenytoin (due to its hydrophobic structure) moves into RBCs and binds to intracorpuscular hemoglobin. When the hemoglobin level is low, the capacity for drug–hemoglobin binding decreases, leading to an increase in unbound phenytoin levels and an increased risk of adverse reactions. However, the possibility that the low hemoglobin level identified in this study is a surrogate for hypoalbuminemia cannot be excluded. Therefore, further extensive investigations of the potential interactions between phenytoin and hemoglobin are required. Additional confirmatory pharmacokinetic investigations are essential for a deeper understanding, particularly in patients with thalassemia or hemoglobinopathy, who constitute up to 40% of our population (Paiboonsukwong et al., 2022; Tepakhan et al., 2024) and in whom shortened RBC lifespans may alter drug-adduct clearance.

The limitations of this study include potential confounding factors, a limited sample size, and a high proportion of missing data. First, most participants in the case group were neurosurgery patients compared to those in the tolerant group. These patients included those with brain tumors or intracranial hemorrhages, who may have had a poorer baseline status than patients with epilepsy, traumatic brain injury, or trigeminal neuralgia. This disparity could confound the lower hemoglobin level due to poorer overall health status or the increased WBC count due to a stress response. However, the relationship between WBC level and susceptibility to drug allergy could be substantial as inflammatory cytokines (specifically tumor necrotic factor-α) could increase the inclination to drug hypersensitivity reactions by overcoming the tolerance of drug-specific autoimmunity (Sun et al., 2025). Second, as a conventional case–control genetic study, this study included a relatively small sample size compared to previous genome-wide association studies. Therefore, the possibility of false-negative and -positive associations with rare genetic markers cannot be excluded. Although a Bonferroni correction was applied, none of the HLA associations remained significant after correction. Accordingly, studies with larger sample sizes are needed to verify our findings. Third, this study had a high proportion of missing data, particularly for laboratory parameters. The baseline clinical chemistry profile was missing for more than half of patients in the tolerant groups, which limited the reliability of the multivariate analysis. There was also a lack of plasma phenytoin-level data (for therapeutic drug monitoring) for all participants. Therefore, it is difficult to assess the independent contributions of genetic and clinical variables. Further validation is required to confirm these findings in different populations. There is also a lack of prospective studies on the pharmacogenetic implementation of phenytoin administration (Jaruthamsophon et al., 2022), and thus further clinical studies on the pharmacogenetic prediction and prevention of PHT-cADRs are necessary.

In conclusion, this study highlights the interplay between genetic and non-genetic factors in the development of PHT-cADRs in a Southern Thai population. The susceptibility to PHT-cADRs is influenced by genetic and non-genetic factors. The association between hematologic parameters and PHT-cADRs constitutes an exploratory observation toward a better understanding of the pharmacokinetics of phenytoin. Therefore, further studies are warranted to refine these predictive models and enhance phenytoin safety.

## Data Availability

The original contributions presented in the study are included in the article. Further inquiries can be directed to the corresponding author.
